# Optimizing Effective Labeling Efficiency in MINFLUX 3D DNA-PAINT Microscopy by Maximizing Marker Detection Probability

**DOI:** 10.1021/acsphotonics.5c01253

**Published:** 2025-12-19

**Authors:** Christian Soeller, Alexandre F. E. Bokhobza, Javier Casares-Arias, Alexander H. Clowsley

**Affiliations:** † Department of Physiology, University of Bern, 3012 Bern, Switzerland; ‡ Department of Biosystems Science and Engineering, Eidgenössische Technische Hochschule (ETH) Zurich, 4058 Basel, Switzerland

**Keywords:** MINFLUX nanoscopy, DNA-PAINT, effective labeling efficiency, marker detection, docking-site stability, super-resolution microscopy

## Abstract

MINFLUX is a powerful single-molecule approach capable of achieving high spatially isotropic resolution in three dimensions. Current implementations collect localizations strictly serially, but criteria for when to terminate acquisition are often unclear. We therefore systematically investigate the time course of effective labeling efficiency (ELE) and achievable saturation values in MINFLUX 3D DNA-PAINT microscopy of Nup96 proteins in a U-2 OS-Nup96-mEGFP modified cell line using a commercial MINFLUX microscope. ELE was measured using a quantitative procedure based on maximum-likelihood template fitting. We collected data measured over various scan sizes and achieved ELE values of ∼60% after passing a time interval dependent on the region size, typically requiring long-duration acquisitions over several hours. Our data and a simple model suggest that maximizing marker detection is key to achieving the limits set by chemical labeling efficiency. A factor limiting the marker detection probability when using conventional DNA-PAINT markers is docking strand site-loss, observed over the duration required to build up the image data of MINFLUX acquisitions, which also limits the achievable number of labeling site visits to values around 1–3. Using repeat DNA-PAINT, i.e., employing oligonucleotide sequences with repeated docking sites, we observed greatly reduced site-loss and could increase the number of individual visits to site locations by more than 3-fold over the same period. Additionally, this enabled increasing stringency criteria for labeling (i.e., higher threshold values) and maximizing marker detection probabilities so that ELE reaches the limits set by chemical labeling efficiency.

## Graphical abstract

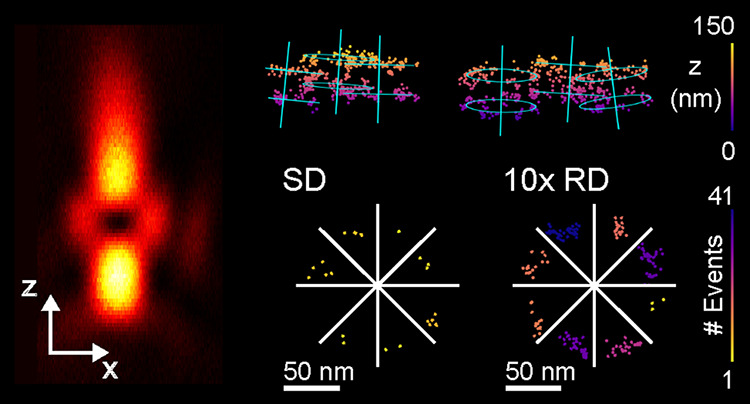

## Introduction

MINFLUX “nanoscopy” is a second-generation super-resolution approach capable of obtaining nanometer precision in three dimensions.^1^ The technique builds upon the foundation of the original super-resolution approaches, STED^2,3^ and STORM/PALM,^4,5^ by combining elements of both. MINFLUX systems rely on rapidly scanning an excitation beam with a known intensity minimum, such as a donut in 2D and a bottle beam pattern for 3D localization, Supporting Figure S1a,b, across a sample attempting to capture single-molecule fluorescence-based signals within the central zero position. One can imagine that, as the beam closes in on its target fewer photons are collected, and the known position of the excitation intensity zero is used to estimate the position of the fluorophore, Supporting Figure S1c. While this intuitive description captures the core principle of MINFLUX, a formal framework that unifies MINFLUX and related methods has been developed, termed ‘single-molecule localization via sequential structured illumination (SML-SSI).^6^ This framework describes localization as a sequential measurement process under structured excitation patterns and provides a rigorous statistical basis using the Cramer-Rao bound for evaluating localization across different localization techniques.

Originally, in single-molecule localization microscopy (SMLM), the generation of individual events was based on photophysical, photochemical or chemical switching of the fluorescent molecular states which imposes strict requirements on the fluorophores suitable for SMLM and often requires special chemical switching buffers to optimally perform.^7^ These dyes are generally permanently attached to the marker being imaged and, as such, eventually photobleach into a nonfluorescent state. To overcome this limitation, approaches using complementary strands of DNA with photostable dyes are becoming more common, termed DNA-PAINT,^8^ and are also being applied to MINFLUX.^9,10^ DNA-PAINT uses dye-modified oligonucleotide sequences, free in-solution, called “imagers”, which are complementary to “docking” strand sites affixed to the target marker.^8,11^ These imagers are free to stochastically, and importantly, reversibly hybridize to an available docking site with a designed mean binding time that is typically a fraction of a second. It is this transient immobilization of the imager that culminates in a detectable single-molecule event. DNA-PAINT, with appropriate excitation parameters and dye choices, circumvents the effects of photobleaching due to the constant replenishment of imagers present within the buffer, however, an alternative loss mechanism termed site-loss has been shown to occur from phototoxicity.^12,13^ In this site-loss scenario, docking strands become chemically modified and accordingly are unable to bind imager stands with the original designed affinity and attachment time. Nevertheless, the versatile approach has enabled the construction of a number of DNA-nanotools for biological investigation at the super-resolution level^14,15^ and innovations have been demonstrated to combat nonspecific interactions in biological samples from both imager^16^ and marker labeling.^17^

In conventional widefield super-resolution microscopy, protein targets in the nuclear pore complex have been introduced as a suitable reference sample to quantify effective labeling efficiency. Quantitative procedures to determine the effective labeling efficiency were established in that study, suggesting values between 40 and 80% can be consistently achieved with suitable high-quality marker systems.^18^ By contrast, MINFLUX effective labeling efficiencies have, to our knowledge, not been previously systematically quantified.

We sought to fill this gap by translating the use of NPC labeling and imaging of Nup96-mEGFP distribution to 3D MINFLUX microscopy to evaluate protocols aimed at maximizing achievable effective labeling efficiency as compared to widefield super-resolution microscopy. Our 3D NPC-based labeling efficiency assays show that MINFLUX DNA-PAINT can achieve similar labeling efficiencies as observed in widefield super-resolution microscopy, but only if care is taken to image for extended durations. Site-loss can be pronounced in MINFLUX DNA-PAINT but is still compatible with achieving effective labeling efficiencies >50%. We demonstrate that site-loss can be effectively mitigated through the use of repeat docking domains, enabling DNA-PAINT experiments to run unaided for hours^13^ thus fully attaining the labeling efficiency limits set by chemical constraints that generally only depend on the marker system in use and the accessibility of the target sites.

## Results and Discussion

### Effective Labeling Efficiency (ELE)

Achieving a high labeling density is critical in super-resolution imaging. In fluorescence microscopy, specifically SMLM, achieving a high effective labeling efficiency involves two major contributions. There is an initial probability of successfully labeling each possible site resulting in the fraction of chemically labeled sites, *f*_chem_, also termed the chemical labeling efficiency, which is influenced by several factors including the density of probes, their accessibility to the labeling target and their binding affinity etc. Once the sample is appropriately labeled to be imaged on a super-resolution setup there is a further contribution from the fraction of those chemically labeled sites that have been detected by imaging, which we term *f*_detect_. *f*_detect_ designates that fraction of chemically labeled sites that generated at least one valid MINFLUX event within a duration T from the beginning of a MINFLUX acquisition series (see also further details in the section on model description in the Supporting Methods). This fraction increases with acquisition duration T until a saturating value is reached, time playing a role due to the, often long, times between visits of an imager to a docking site. In a best-case scenario, all chemically labeled sites are ultimately detected, with *f*_detect_(*T*) approaching 1 for long acquisition duration T. To detect a chemically labeled site in MINFLUX microscopy at least one stochastic single-molecule emission event, coinciding with the scan location, overcoming the acquisition detection criteria (implicit in the MINFLUX acquisition “sequence” definition, see Methods and Supporting Figure S2) must be registered. In SMLM and specifically in MINFLUX microscopy, many images and/or events are sequentially detected over an extended time, accordingly *f*_detect_(*T*) generally increases with imaging duration T. Eventually, a limiting maximal value is reached that in DNA-PAINT may be influenced by site-loss/marker damage from reactive oxygen species photodamage. Together, the labeling and detection fractions determine the effective labeling efficiency ELE­(*T*) = *f*_chem_ *f*_detect_(*T*) which increases with acquisition time T until the fraction of detected sites saturates. Here we use a prototypical labeling system, using by now well-established single-domain antibodies (sdAB) against GFP, and modified with docking strands for DNA-PAINT, to label Nup96-eGFP in a U-2 OS cell line and investigate how to maximize the detection probability in MINFLUX imaging, while having an essentially unaltered chemical labeling efficiency, *f*_chem_, across experiments. Using the NPC-based labeling system we measure the effective labeling efficiency with MINFLUX DNA-PAINT in 3D while varying imaging parameters and identify conditions to maximize the detected fraction of chemically labeled sites (*f*_detect_) with the goal that ELE should approach *f*_chem_ in the best-case scenario.

### Imaging of the Nuclear Pore Complex (NPC) as a Convenient Biological Standard

We opted to use the CRISPR-engineered U-2 OS cell line endogenously expressing GFP on Nup96 proteins as they provide a well-characterized biological structure with, effectively, a well-known protein arrangement.^18^ With a diffraction limited confocal scan of the MINFLUX microscope, the Nup96 protein locations are not resolved, Figure 1a. Instead, one sees a collection of essentially featureless puncta, where each punctum of GFP signal encompasses the position of a whole NPC, enabling a general initial assessment of the nature of the cell (inset panel Figure 1a). Only once super-resolved, for instance from DNA-PAINT localizations (see “blinks” recorded in the confocal mode of the MINFLUX microscope, Figure 1bi) do Nup96 locations become more apparent, as can be seen in two views of a single NPC structure recorded as part of an extended duration MINFLUX acquisition (Figure 1bii,iii).

**Figure 1:**
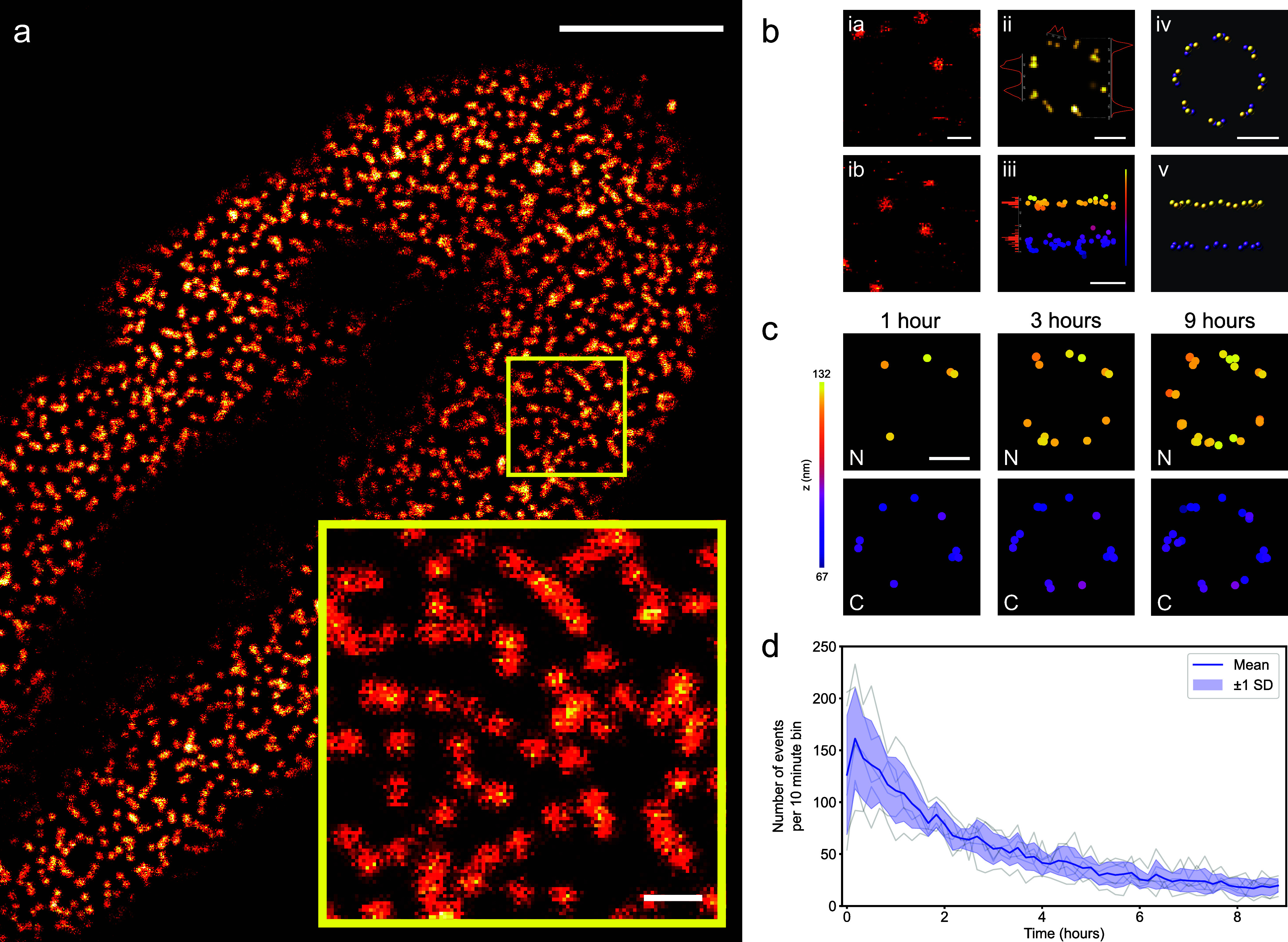
MINFLUX super-resolution imaging of NPC proteins. (a) A confocal overview image of a U-2 OS-Nup96-mEGFP modified cell. The zoomed-in image of the ∼3 × 3 μm^2^ boxed region shows the mEGFP diffraction-limited signal displaying the positions of nuclear pore complexes (NPCs). (b). i­(a,b): Frames from fast confocal scans showing the transient attachment of imagers to markers on Nup96-mEGFP; ii, iii: *x*–*y* and *x*–*z* views of MINFLUX localizations associated with a single NPC structure; iv, v: schematic of the arrangement of Nup96-mEGFP targets. (c) Example MINFLUX localizations of Nup96 proteins from nucleoplasmic (N) and cytoplasmic (C) NPC rings at different points in time. As the scan time increases the completeness of the Nup96 distribution for both rings improve with some sites obtaining additional revisits. (d) Over the time course of acquisition the MINFLUX event rate decays successively, here shown for MINFLUX regions measuring 3 × 3 μm^2^ (*n* = 6 MINFLUX data sets from 4 replicates). Scale bars: (a): 5 μm, inset: 500 nm, (b) ia and ib: 500 nm and ii–iv: 50 nm, (c): 50 nm.

The MINFLUX localizations reflect the structure of the Nup96 site distribution, where, in the plane of the nuclear envelope, individual Nup96 proteins are spaced ∼12 nm apart between pairs and each separated ∼42 nm from their closest neighboring pair making a symmetrical ring of eight, as shown in the schematic Figure 1b­(iv). This pattern is replicated on both sides of the nuclear envelope to give essentially two “rings” on both the nucleoplasmic side (N) and the cytoplasmic side (C) separated by ∼50 nm (Figure 1b­(v)). In total, 32 Nup96 proteins can be labeled per NPC. As in other SMLM methodologies, MINFLUX microscopy builds an image gradually over time. In current implementations of MINFLUX microscopy we have the extreme case that one molecule is localized at a time and all localizations are acquired strictly sequentially with no parallel processing (which helps accelerate camera-based widefield SMLM). Accordingly, structures such as the NPC structures imaged here, become gradually more complete as an acquisition progresses, as shown in Figure 1c.

In MINFLUX 3D DNA-PAINT, we observe a fairly pronounced decay of localization rates over the time course required to build up the structure (Figure 1d) and this decay appears to result from site-loss, as we show further below. Due to the inherently serial nature of the acquisition in single-point scanning approaches like MINFLUX, the time required to sufficiently sample a specimen is expected to be longer than in widefield single-molecule acquisitions using a camera, where many emitters can be captured simultaneously. To quantify these qualitative observations, we established a refined version of the approaches in Thevathasan et al.^18^ to more precisely measure effective labeling efficiency, here specifically tailored to the 3D MINFLUX microscopy modality.

### MINFLUX DNA-PAINT 3D Imaging of NPCs and Quantification of ELE

#### Titrating Imager Concentrations for DNA-PAINT

The rate of single-molecule events in DNA-PAINT is dependent on several factors, including: the number of available docking sites on a marker, the density of the markers, the imager concentration, and the buffer composition. Prior to MINFLUX experiments, we therefore took short *xyt* confocal scans with set parameters (see also Figure 1bi and Methods section), to ascertain appropriate imager blink rates. These scans enabled us to qualitatively assess the level of imager to docking-site bindings and reduce the likelihood of overlapping events by titrating the imager concentration, see also Supporting Media M1. This is especially important in MINFLUX microscopy as such overlaps can lead to the failure of MINFLUX localizations and/or localization errors.^19^ Conversely, the imager concentrations should be high enough to achieve a high rate of successful localizations to keep acquisition times in a reasonable range.

Prior to NPC labeling analysis, localizations from the same trace were coalesced to a single detection at the centroid of the “trace” based on the inherent “traceID” property, with lower localization error resulting from coalescing, as described previously.^9,10^ All events in the same trace will originate from a single imager binding to a single docking strand and should therefore be treated as a single “compound localization”.

#### Maximum-Likelihood Fitting of NPC Structures

To estimate ELE, we used an approach similar to the method introduced by Thevathasan et al.,^18^ but extended to 3D for MINFLUX microscopy. MINFLUX 3D data of Nup96-mEGFP labeled with anti-GFP DNA-PAINT sdABs (Figure 2a) were rendered to a 2D image in PYMEvisualize and locations of NPCs were detected by a semimanual approach in Fiji^20^ and stored as regions-of-interest (ROIs) in ImageJ ROI format. ROIs were read into the PYMEvisualize^21^ SMLM data visualizer and used to label coalesced 3D MINFLUX localizations belonging to identified NPCs with a unique ID. We then used maximum-likelihood fitting to a “double ring” template to align NPC events with the template using an algorithm as described,^22^ which we implemented into PYMEvisualize.^21^ Following maximum-likelihood fitting, NPC events were generally well-aligned with the template (Figure 2b). The transformed coordinates are used for further determination of labeling of NPC segments in the two rings. In addition, the optimal fitting parameters are also used to display a suitably rotated template schematic overlaid with the original MINFLUX localizations in PYMEvisualize, see Figure 2c-i,ii,iii. In addition to the two rings, the overlays contain the central symmetry axis of each NPC structure and reveal the variation in NPC orientation across the field of view, presumably reflecting variations in the plane of the nuclear envelope.

**Figure 2:**
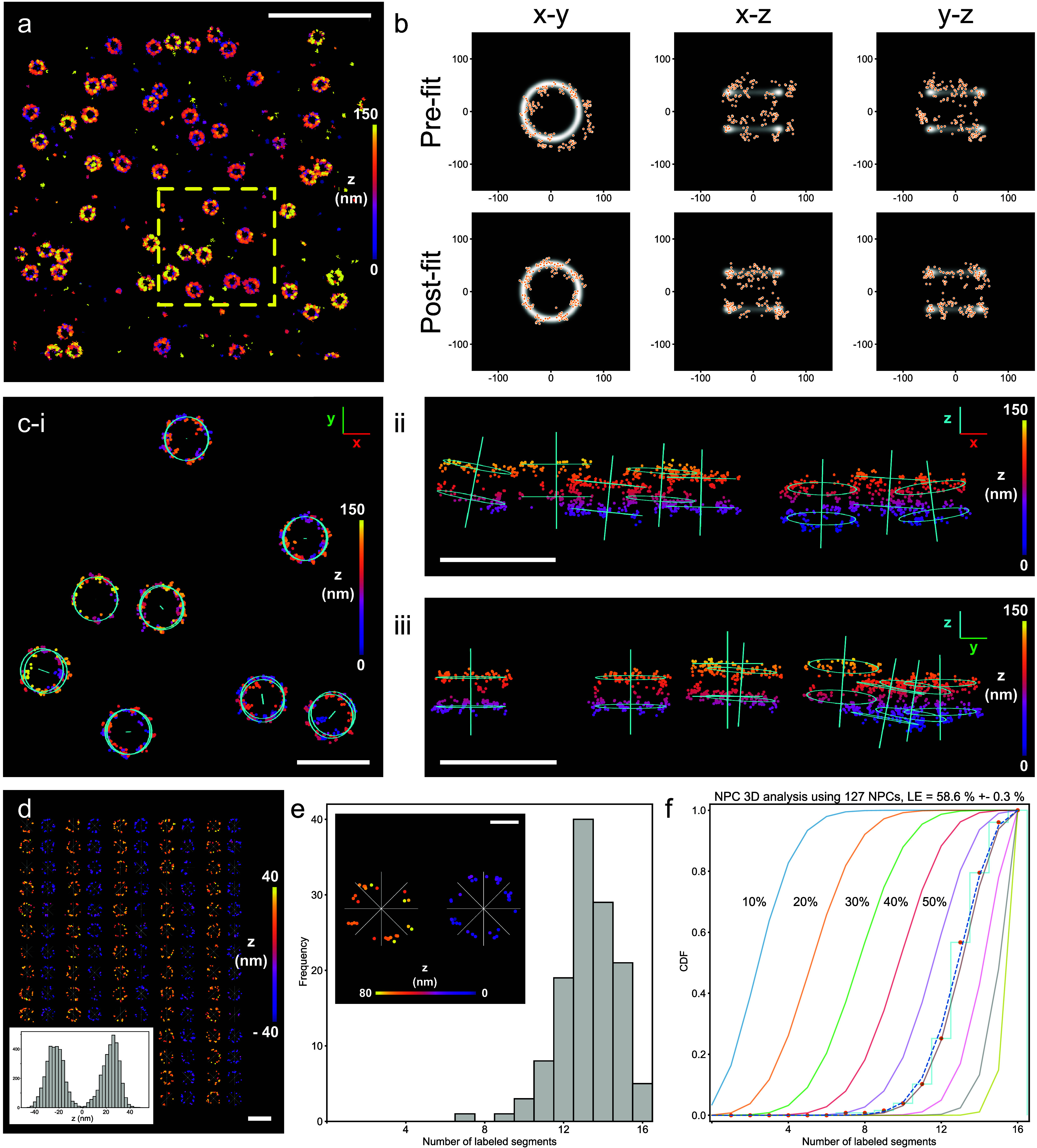
3D NPC labeling efficiency methodology. (a) A typical region of U-2 OS-NUP96-mEGFP NPCs localized using a MINFLUX microscope. (b) Individual NPC localizations, shown in *x*–*y*, *x*–*z* and *y*–*z*, displayed over a double ringed template structure for both prefit and postfit localizations whereby transformations were applied to achieve the best fit with the template. (c-i) Fitted NPCs, taken from the boxed region in (a), show the additional geometry from the points fitted to the rings in *x*–*y*. Axial visualization, (c-ii): *x*–*z*, and (c-iii): *y*–*z* demonstrate the subtle curvatures of the nuclear envelope, indicated by the axis along the pore which was added to the template overlay for clarity. (d) A gallery representation of the nucleoplasmic and cytoplasmic rings, for individual NPCs, displayed next to one another enables a quick assessment of the quality of the data. A histogram of these NPCs exhibits an approximate 50 nm peak to peak separation in *z*. (e) A histogram showing the number of NPC segments labeled for the data set. The localizations are separated into 8 segments for both nucleoplasmic and cytoplasmic NPC rings, see inset. (f) Cumulative distribution function plot of labeled segments (data, red dots) and estimated effective labeling efficiencies based on the model fit (dashed line). Solid lines show model predictions for 10–90% ELE. Color maps indicate localized position in *z*. Scale bars: (a): 1 μm, (c-i, c-ii and c-iii): 200 nm, (d, e): 50 nm.

#### Histograms and Fitting with a Model Prediction

Figure 2d shows a “gallery” of all fitted NPC structures from one MINFLUX 3D data set, separated into nucleoplasmic (orange/yellow) and cytoplasmic (blue/violet) components, side by side, displaying the axial separation of the contribution from the two sides at the expected distance of ∼50 nm (Figure 2d, inset). After alignment to “segment boundaries”, all fitted NPC localization data sets were also rotationally aligned (Figure 2e, inset). A selectable threshold (typically a minimum of 1 or 3 events per segment; see threshold determination in Supporting Figure S3) was then used to determine whether a segment was labeled, separately for nucleoplasmic and cytoplasmic sides. This was used to construct histograms (Figure 2e) showing, for the NPC data set, the number of segments labeled (from 0 to 16). The histograms were converted to cumulative form and, in this form, plotted against the predicted model behavior (showing model curves for 10 to 90% labeling as solid curves), with the model constructed based on the assumption of independent labeling at all 32 Nup96 sites (see Supporting Methods). A value of best fit to the model was then determined. The procedure thus culminates in providing this value of best fit, in the sample shown in Figure 2f obtaining a value of *P*_ELE_ = 58.6 ± 0.3% for a large ROI data set containing 127 NPC structures. We proceeded to use this approach to determine the time course and limiting value of *P*_ELE_ for different MINFLUX region sizes using conventional DNA-PAINT docking strands.

### ELE with Conventional DNA-PAINT

As an additional consequence of MINFLUX being a single-point scanning system, the size of the region selected for imaging has a direct impact on the time to acquire a “complete” image. We therefore measured the ELE of U-2 OS-Nup96-mEGFP NPCs at various time points across several scan region sizes we term “small”, “medium“ and “large”, corresponding to ROI areas of approximately 2, 9, and 25 μm^2^ and encompassing around 10, 50, or 100 visible NPCs, respectively. Data was generally acquired for extended time periods, over several hours, and we analyzed subsets of the data with increasing analysis cutoff time *T*_max_ by filtering only for events with a time of localization *t* ≤ *T*_max_ in the PYME analysis environment to determine how ELE increases progressively with time.

Within the first hour, using the smallest ROIs, an ELE of 55.9 ± 13.0% was obtained, compared to 32.7 ± 9.5% for the medium size ROI, and 17.4 ± 2.5% for the largest field of view imaged, Figure 3a (all values are reported as mean ± standard deviation for all NPCs measured, see also Supporting Table T1). With MINFLUX DNA-PAINT, the small ROI achieved the highest level of *ELE* after ∼3 h, 68.2 ± 9.0%, whereas the medium and large ROIs peaked slightly lower with means of 61.4 ± 5.7 and 54.6 ± 3.5% obtained within 9 and 12 h of recording, respectively (although changes in ELE were not significant for the large ROIs beyond 6 h). The difference between the final ELE values for small and medium regions and between medium and large regions were also not statistically significant. If experiments are continued beyond these durations, ELE does not increase statistically any further (see, for example, the large ROI results in Figure 3a). This is due to two effects, (1) revisits of the same sites do not increase ELE further, and (2) although DNA-PAINT overcomes photobleaching of imagers, it does still experience a lowering of localization rates due to site-loss.

**Figure 3:**
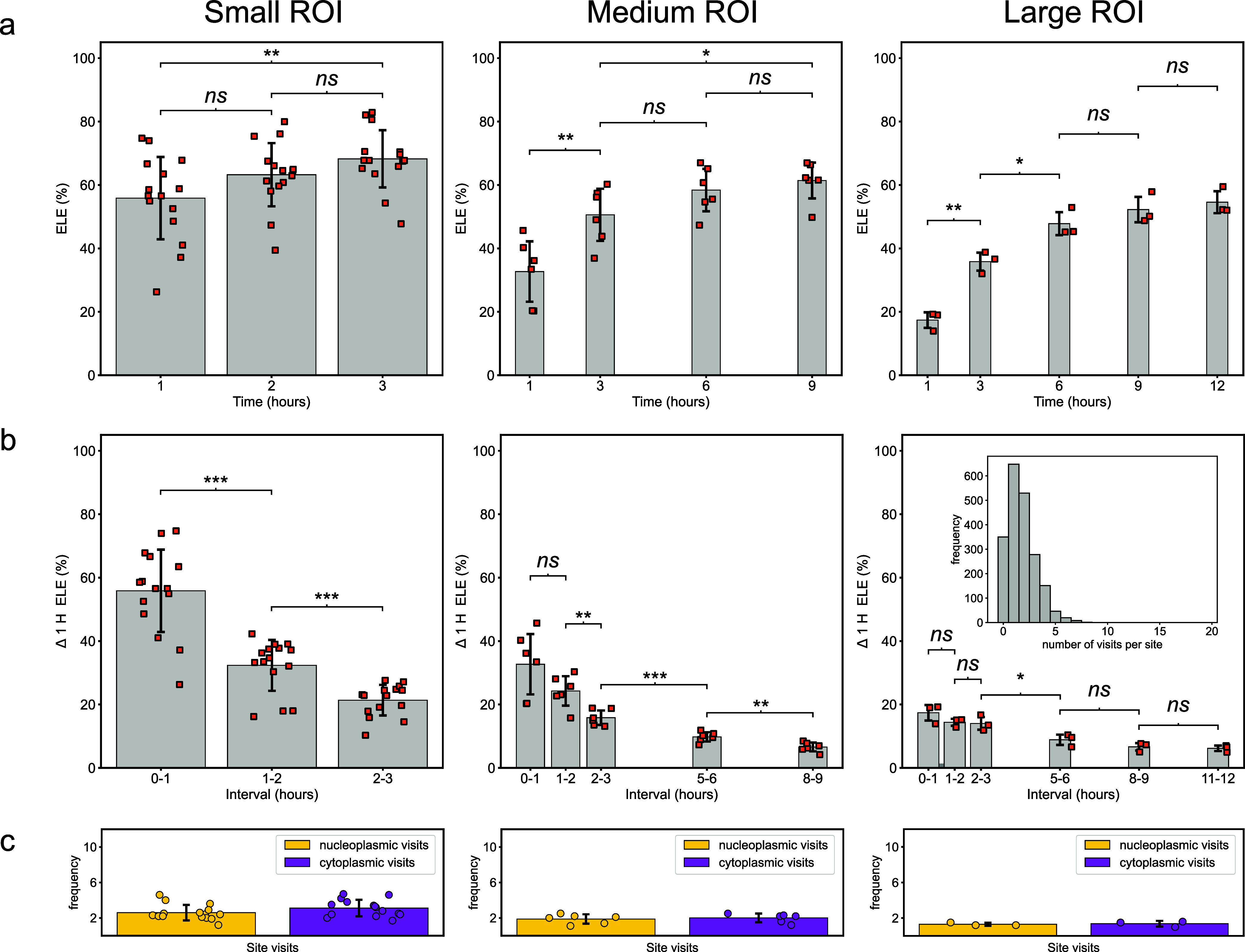
Effective labeling efficiency (ELE) over time. (a) Calculated ELE over time for increasing MINFLUX scan-size regions, from small to large, incorporating approximately 10, 50, and 100 NPCs per experimental condition. (b) ELE measurements taken at 1 h intervals for the same NPCs in (a) demonstrate substantial DNA-PAINT site-loss as the experiment progresses for all scan-sizes. A histogram inset within the data for the “large” ROI demonstrates how the site-loss results in only a small number of revisits to DNA-PAINT marker sites in MINFLUX experiments. (c) Bar graphs show the number of visits to both nucleoplasmic and cytoplasmic sites for each of the imaging fields of view obtained at their maximal ELE time points: 3, 9, and 12 h. These data were collected from N independent repeats, *n*_cell_ imaged cells, and *n*_NPC_ imaged NPCs. Small ROI: *N* = 4, *n*_cell_ = 15, *n*_NPC_ = 209, medium ROI: *N* = 4, *n*_cell_ = 6, *n*_NPC_ = 280, large ROI: *N* = 3, *n*_cell_ = 3, *n*_NPC_ = 342. A *p*-value <0.05 was considered statistically significant and denoted as *, *p* < 0.01 as **, *p* < 0.001 as ***, and nonsignificance as *ns*. Scale bar: 50 nm.

This effect of site-loss becomes clear by examining how ELE was affected by restricting the data used for ELE analysis at hourly intervals as the experiments progressed, in each case only using data from 1 h, at analysis starting times of 1, 2, and 3 h since MINFLUX acquisition began (and several later times with medium and large ROIs), Supporting Table T2. For all ROI sizes, we observed a progressive decrease in the ELE as compared to measurements taken within the first hour, Figure 3b, which reflects progressive site-loss as also seen by the gradual reduction in event rates (compare also Figure 1d).

Site-loss has an important effect by limiting the number of revisits of individual target sites. We investigated this by measuring the average number of MINFLUX localizations within a single segment of an NPC that contains two Nup96 target sites (see also schematic in Figure 1biv,v). Site visit histograms are typically unimodal, right-skewed distributions with a small tail resembling an exponential decay, with many sites only visited a single time, especially for medium- and large-sized ROIs, as illustrated in the inset of Figure 3b. On average, after an acquisition time at which ELE becomes maximal (3, 6, or 9h for small, medium and large ROIs, respectively) 2.6/3.1 ± 0.9/0.9 (nucleoplasmic side (N) /cytoplasmic side (C), small ROI), 1.9/2.0 ± 0.5/0.5 (N/C, medium ROI) and 1.3/1.4 ± 0.2/0.3 (N/C, large ROI) visits per site were achieved, as shown in Figure 3c. Importantly, these numbers do not appreciably increase by imaging for longer due to irreversible site-loss (see, for example, Supporting Figure S4).

Finally, we briefly compared these results with some data from MINFLUX dSTORM experiments. In contrast to MINFLUX DNA-PAINT, in photoswitching MINFLUX dSTORM acquisitions with Alexa Fluor 647, a much smaller ELE at the end of experiments (dictated by the near complete decay of localization rates) of 29.5 ± 2.3% was obtained (for medium-sized ROIs, Supporting Figure S5a, see also Supporting Table T3). This coincided with a rapid decrease in the number of localizations detected as the scan proceeded, Supporting Figure S5b. Note that the decay of localization rates can be greatly influenced by the makeup and performance of the inherently labile switching buffers, therefore precise dSTORM results may differ across laboratories.

### ELE with Repeat DNA-PAINT

Our analysis of MINFLUX data of NPCs with conventional DNA-PAINT docking strands suggests that site-loss may preclude further increasing the detection fraction *f*_detect_. In addition, from a spatial resolution point of view and to increase confidence in imaging results in SMLM, it is generally desirable to increase the number of site visits at a target site. A promising approach in this context is repeat DNA-PAINT, i.e., using DNA-PAINT markers with multiple repeat docking domains,^13,23,24^ shown in the schematic in Figure 4a, as established by us and others. Composite rendered images constructed by averaging multiple NPCs for both single-domain (SD, Figure 4ai) and repeat-domain (RD, Figure 4aii) exhibit clustering suggestive of resolving the 12 nm spacing between individual sites. Markers bearing repeat-domains have a number of advantages, including the ability to reduce imager concentrations and accelerate acquisition. Here, we decided to utilize a 10× RD as we had previously shown that this configuration can greatly reduce the rate of site-loss while having a negligible impact on achievable resolution due to the random coil behavior of single-stranded DNA.^13^ Inspecting the number of MINFLUX event localizations over time, we observe a near constant rate of detection for the repeat-domain in comparison to those from single-domain experiments, Supporting Figure S6. In comparison to a marker exhibiting a single-domain docking strand, the repeat strands were operated at an approximate order of magnitude reduced imager concentration, which also helps reduce background fluorescence from imagers in solution. When imaged for an extended period of time, ∼9 h, the repeat-domain markers achieved ∼3 times as many visits per site for the equivalent MINFLUX imaging duration, Figure 4b. Despite this, we interestingly found Fourier Shell Correlation (FSC) measurements, a common resolution assessment for super-resolution and electron microscopy images which compares the correlation between two images at different spatial frequencies in Fourier space,^25^ for SD and RD to be similar to one another at around 9 nm, Supporting Figure S7. When evaluated with an increased threshold of at least 3 events in a segment (enabled by the much higher number of target site visits), the small ROI gave an ELE of 35.4 ± 14.9% within the first hour, by 3 h this had matched the single-domain lower threshold at 68.4 ± 7.7%. Similar results were observed with a medium-sized ROI, 63.9 ± 11.2% at 9 h, Figure 4c, see Supporting Table T4. See also Supporting Figure S8 and Table T5 to compare RD data at the lower threshold.

**Figure 4:**
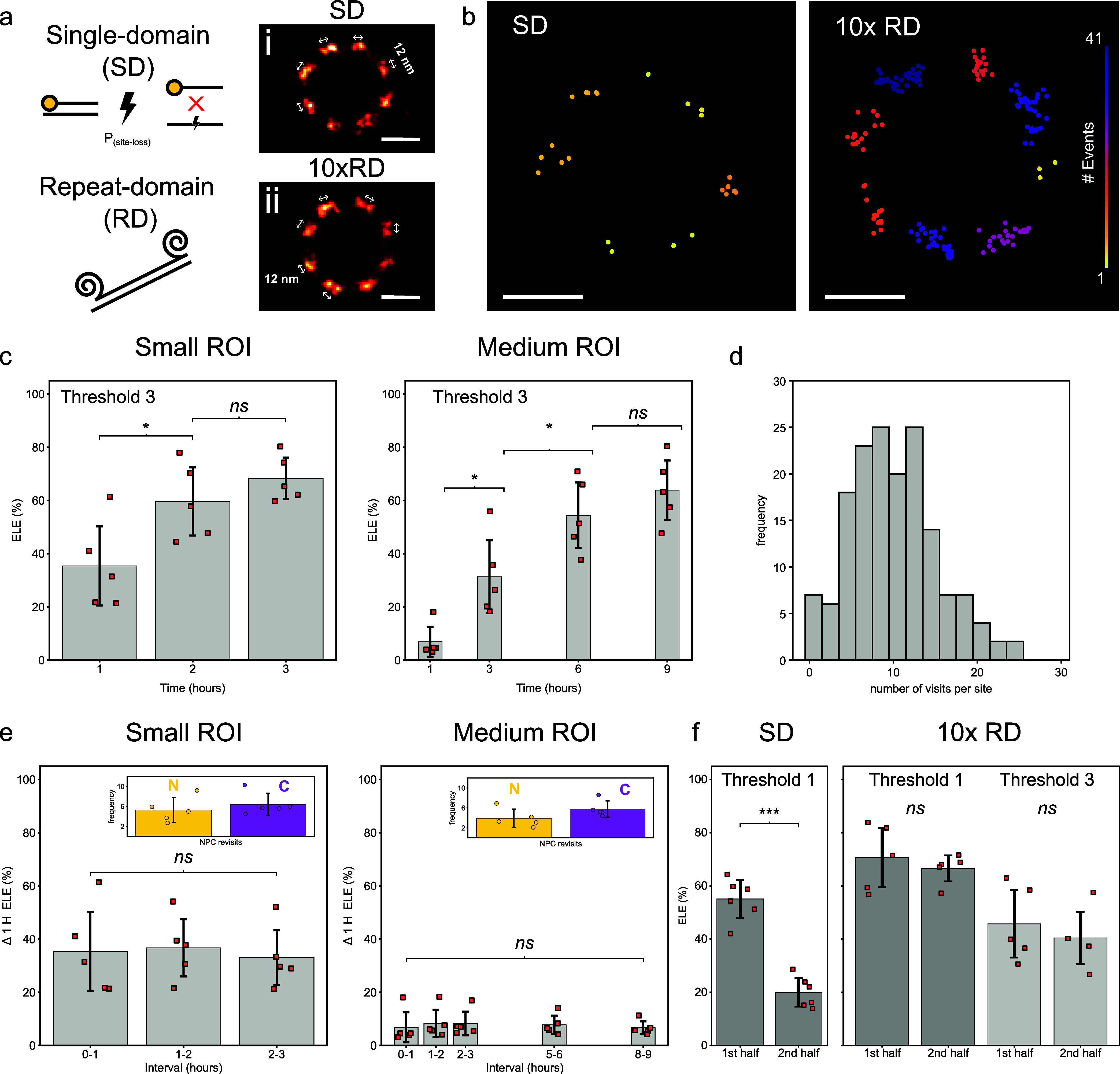
Repeat-domain (RD) ELE. (a) A schematic depicting the susceptibility for DNA-PAINT docking strands to become photodamaged during a MINFLUX acquisition. An oligonucleotide consisting of repeat docking sequences fixed to target DNA-PAINT markers provides additional redundancy, increasing the probability of detection as an experiment progresses. Gallery rendered images for (i) single-domain (SD) and (ii) 10× RD averaged NPC images using template aligned NPCs from two exemplary small ROI data sets (b). The additional docking locations results in a greater number of revisits to Nup96 sites, approximately 3-fold. Example localizations from cytosolic NPC rings exhibit a greater number of returns for similar scan sizes at the same time point. (c) ELE for 10× RD for both small and medium scan sizes using an increased threshold requiring a minimum of three visits per site (d). A typical histogram of site visits in this configuration no longer exhibits the near-exponential behavior observed with single-domain site-loss. (e) ELE measurements taken at 1 h intervals for the same NPCs in (c) demonstrate the effect of having additional redundancy to reduce the observable effects of DNA-PAINT site-loss during MINFLUX imaging. This additionally assists the number of revisits each site obtains, see inset bar graphs for nucleoplasmic (N) and cytoplasmic (C) visits at 3 and 9 h for the “small” and “medium” ROIs. (f) ELE measurements for the first and second half of experiments examining a “medium” ROI for normal DNA-PAINT harboring a single-domain (SD) demonstrate a significant reduction in the obtainable ELE. Whereas RD DNA-PAINT demonstrates a considerable resistance to site-loss for the same experimental conditions. These data were collected from N independent repeats, *n*_cell_ imaged cells, and *n*_NPC_ imaged NPCs. Small ROI: *N* = 3, *n*_cell_ = 5, *n*_NPC_ = 61, medium ROI: *N* = 3, *n*_cell_ = 5, *n*_NPC_ = 177, and for the SD medium ROI: *N* = 4, *n*_cell_ = 6, *n*_NPC_ = 280. A *p*-value <0.05 was considered as statistically significant and denoted as *, *p* < 0.01 as **, *p* < 0.001 as ***, and nonsignificance as *ns*. Scale bars: 50 nm.

A typical histogram of site visits in this configuration has a mode at 10 site visits (Figure 4d) and has lost the near-exponential appearance resulting from substantial site-loss with conventional DNA-PAINT. In agreement with the idea that site-loss is greatly reduced with this strategy, the measurement of ELE at hourly intervals revealed a steady state of Nup96 detections in both small (35.1 ± 2.1%) and medium (7.6 ± 1.2%) ROIs, Figure 4e, see also Supporting Table T6. Examination of the mean number of localized events per NPC demonstrated a linear relationship for the repeat-domain experiments, indicating minimal site-loss compared to those from single-domain, Supporting Figure S9. The number of site visits increases with repeat DNA-PAINT to 5.3/6.4 ± 2.5/2.2 (N/C, small ROI) and 3.9/5.8 ± 1.8/1.6 (N/C, medium ROI) after 3 and 9 h, respectively (Figure 4e, insets). Interestingly, with the increased revisits per site, our repeat-domain acquisitions had a slightly elevated number of cytosolic event detection compared to those closer to the nucleus, see also Supporting Figure S10.

When data sets were split in half by acquisition time, the ELE achieved using localizations from the first and second part could be compared. Single-domain (SD) DNA-PAINT exhibited a stark contrast to 10× RD experiments with a reduction in ELE 55.1 ± 7.2 to 20.0 ± 5.3%, *p* ≪ 0.001, whereas for 10× RD ELE remains statisticaly indistinguishable at 45.7 ± 12.7 vs 40.4 ± 9.9%, *p* = 0.528 (Figure 4f). Note that with a lower threshold of 1, this increases to a 10x RD ELE of 70.7 ± 11.1 vs 66.6 ± 4.9, *p* = 0.521.

As we improve the obtainable resolution of our microscopy approaches, it is essential to consistently benchmark the quality of the data produced. MINFLUX is a powerful technique, but it requires a complex implementation. With the use of the U-2 OS-Nup96-mEGFP modified cell line we have benchmarked the obtainable ELE using DNA-PAINT single-molecule events on a commercial MINFLUX microscope system. The results and open-source software will enable other users to check the quality of their labeling procedures and determine their individual detection capabilities.

The Nup96 protein is an ideal biological target for quantitative assessment of labeling and experimental conditions, as has been previously outlined.^18^ As we show here, in MINFLUX experiments, not only the chemical labeling capabilities but also the detection probability play a critical role in the obtainable data. Our measurements over various MINFLUX scan-size regions demonstrated how easily one can under-sample a super-resolution target by simply stopping the experiment too early. Using a known biological structure was critical in assessing this. The data we have acquired to date and the measurements shown here lead us to favor DNA-PAINT over the conventional single-molecule direct photoswitching of dyes like Alexa Fluor 647 to avoid the labile nature of the photoswitching buffers necessary for lasting blink performance.^7^ In our hands, the dSTORM-type MINFLUX experiments were generally brought to an end prematurely as the fluorophores enter states of permanent photobleaching, leading to low ELE values for even moderate scan sizes. dSTORM-based MINFLUX experiments may be able to achieve similar high ELEs with virtually complete marker detection by using optimized buffers and imaging settings. However, by comparison, the DNA-PAINT approach should be more easily replicable across laboratories and, in our experience, efficient marker detection can be robustly achieved by moderately experienced users.

Nevertheless, in MINFLUX DNA-PAINT experiments we also observed a drop in the number of detected events as the experiment proceeded that are most likely attributable to site-loss. This is an effect that we and others have mostly studied in synthetic samples where there is known to be a single docking site at the marker location.^12,13^ Custom DNA-PAINT IgG secondary antibodies with unknown (typically 1–3) quantities of docking strands and amplification resulting from a primary/secondary AB labeling system, provide some redundancies to photodamage and, in widefield super-resolution setups, have been demonstrated to produce near constant event rates.^26^ We attribute the noticeable site-loss, observed in our ‘normal’, single-domain DNA-PAINT docking strand MINFLUX experiments, to the serial nature of the scanning MINFLUX approach, combined with the use of single-domain nanobodies that exhibit only a single docking strand per marker. With this labeling approach, as a DNA-PAINT experiment progresses over time, the likelihood of returning new data points diminishes. This means that it takes longer to reach the maximum obtainable ELE values, and in the worst case, where all sites become damaged and no new additional events are possible, the detection probability never reaches 100%, meaning that the upper limit ELE value set by the chemical labeling efficiency is never reached.

To interpret our results and guide the design of MINFLUX experiments we constructed a very simple phenomenological model that, while clearly neglecting much photophysical detail, recapitulates our main findings. The model is described in the supporting methods and its main behavior is illustrated in Supporting Figure S11. It uses primarily two time constants, where τ_revisit_ is the time between imager visits to a site and τ_SL_ is a site-loss time constant. Given the limitations of the simple model (see Supporting Methods) its primary usefulness is that (a) one can relate these two time constants with the time-course over which ELE saturates and site-loss occurs (b) use the insight that the ratio of α ≈ τ_revisit_/τ_SL_ is useful to predict the qualitative behavior of the progressing MINFLUX DNA-PAINT acquisition. Specifically, the model recapitulates that the detected fraction increases over time as a saturating exponential with a time constant that is always faster than the site-loss time constant τ_SL_. More importantly, the saturating value of the detected fraction can be estimated from the ratio τ_revisit_/τ_SL_, with increasing site-loss it may be well below 1, predicting a limiting fraction *f*_max_ = (1 + τ_revisit_/τ_SL_)^−1^. The time constant of ELE saturation, termed τ_eff_^′^, is accelerated as compared to τ_revisit_ by the same factor *f*_max_, see also Supporting Figure S11. From experimental data *f*_max_ can be estimated as 1 – τ_eff_^′^/τ_SL_ i.e., using only the time constants of ELE saturation and site-loss. Using this approach, for example, for the “large” ROI with half-times of ELE saturation ∼2 h (see Figure 3a) and the half-time of site-loss 5.2 h (Supporting Figure S6), *f*_max_ ≈ 0.62. With ELE saturating at ∼0.5, this estimates *f*_max_ = ELE/*f*_max_ as ∼0.8, close to the values estimated for negligible site-loss.

In addition, the site visit histograms from the simple model recapitulate the qualitative transition we observe in the experiments, from an exponential-like behavior with 1 or 2 site visits at most to repeated visits with reduced site-loss in repeat DNA-PAINT.

We note that experimentally we always measure ELE that is the product of chemical and detection fractions, *f*_chem_ *f*_detect_. In good approximation, *f*_chem_ should be constant across experiments, so that we can interpret these to show ELE = *f*_chem_ *f*_detect_, with a constant *c*_chem_ that is in the range 0.7–0.85,^18^ in agreement with the estimate above. As one might expect, as the scan size area increases the time required to reach an equivalent ELE measurement is also increased proportionally. Our results corroborate this assumption, suggesting that for the larger scan sizes the 12 h may possibly not be long enough. This imaging duration for a single data set is becoming practically prohibitive and we believe that our findings suggest that, with the current implementation using a single scanning spot, it is preferable to acquire several (to increase sampling) smaller ROIs to saturation (1–2 h) rather than taking one large ROI requiring over an order of magnitude longer, i.e., ∼10–20 h. In approaching MINFLUX experiments in this manner it also avoids potential experimental issues during a single long acquisition that may affect the data set such as (inevitable) software bugs, uncorrected drift, or changes to buffer composition to name a few. In agreement with this general observation, the number of site visits is also larger with small ROIs, which is desirable in improving confidence in the distribution of localized events. MINFLUX standard DNA-PAINT experiments with an ATTO 655 imager in standard buffer exhibit clear indications that site-loss occurs during an acquisition (Figure 1d, and Supporting Figures S6 and S9) and may additionally contribute to the lower ELE values we observe with larger ROI sizes. In this regard, we believe a future systematic investigation could be useful to obtain greater understanding of these effects by using an approach similar to the in-silico MINFLUX evaluations recently published.^19^

Overall, the substantial site-loss we observed with standard DNA-PAINT was only rectified by using repeat DNA-PAINT which enabled sustained acquisitions with many site revisits. The mean number of revisits can be adjusted by altering the acquisition duration. In addition, repeat DNA-PAINT allows increasing detection fraction *f*_detect_ close to 100%, exploiting the achievable limit set by chemical labeling.

It is possible that other properties of the scan or the sample affect site-loss, such as the effective chemical labeling density. These may influence the ratio of effective revisit times and characteristic site-loss times. For this reason, experiments to measure the time course and maximize *f*_detect_ should be carried out for the biological sample in question. Due to the purely serial nature of current MINFLUX implementations this requires extensive imaging durations, even for moderate ROI sizes.

Our repeat-domain sequences provide 10 identical binding locations for the imager to bind. This feature provides redundancy if any one site becomes damaged during a MINFLUX localization or by being in proximity to one. This is a potential advantage over shorter sequence designs that utilize alternating nucleotides because if one nucleotide is damaged it may affect several “overlapping” docking domains.^27^ With our 10× RD, we observed a near-constant event rate, also indicated by the measurement of ELEs at hourly intervals, suggesting that fewer repeat-domains might be sufficient in practice, thereby reducing manufacturing costs. The repeat-domains additionally enable the user to reduce imager concentrations, consequently reducing the effects of backgrounds which should be beneficial^19^ while maintaining binding kinetics, and is also compatible with a fluorogenic approach.^16^ Other techniques could be used to reduce site-loss, either in combination with repeat DNA-PAINT or as alternatives. Imager dye stability may be a factor since it is likely that excited dye molecules that enter radical states are responsible for site-loss.^12^ Cyclooctatetraene (COT) can quench triplet excited states via photophysical mechanisms, such as through triplet–triplet energy transfer (TET) and dissipates the energy leading to fewer reactive oxygen species.^28–30^ Recently, the use of COT modified oligonucleotides or simply the addition of COT in imaging buffers were demonstrated to reduce the effects of photodamage in DNA-PAINT and could be combined with single- or repeat-domains to further enhance MINFLUX data acquisitions.^31^ It seems difficult to reduce site-loss by reducing illumination intensity as sufficient intensity is needed to ensure a high event rate and localization precision; Ostersehlt et al.^9^ recommend at least 62 μW for the first MINFLUX sequence iteration (see Supporting Information therein) which is comparable to the intensity that we used (∼65 μW). Therefore, using more photostable dyes or solution additives to reduce triplet state lifetimes may have a useful effect.^32^

## Conclusion

The ELE values we report are comparable to previously reported values using other super-resolution approaches and can be used to quantify other MINFLUX, and similar, experimental setups. The ELE determination reported here could additionally be used in cocultured samples to quantify the ELE of other GFP-tagged targets by fixing and labeling cells together on the same slide.

## Methods

### Sample Preparation

#### Oligonucleotides

Tag X2 anti-GFP single-domain nanobodies were purchased from Massive Photonics in either their default “Docking Site 3” and “Imager 3” configuration with undisclosed sequences or as a custom conjugation to the D2 sequence. The repeat-domain (10× RD P1) and P1 8nt imager with a 3′ ATTO 655 dye modification were ordered from Eurofins and were HPLC purified. See Table 1 for the oligonucleotide sequences used. The D2 design was previously used to functionalize markers for repeat docking domains^13^ and has additionally been used for other applications.^14,17^

**Table 1:** Sequences Used for Repeat DNA-PAINT

sequence name	sequence (5′ → 3′)	3′ mod
D2	TTT TAG GTA AAT TTT GAT TGT GAG GAA G	
P1 imager (8 nt)	AGA TGT AT	ATTO 655
10× RD P1	ATA CAT CTA ATA CAT CTA ATA CAT CTA ATA CAT CTA ATA CAT CTA CTT CCT CAC AAT CAA AAT TTA CCT AAC ATA CAT CTA ATA CAT CTA ATA CAT CTA ATA CAT CTA ATA CAT CTA	

#### Cell Culture

U-2 OS-Nup96-mEGFP and Nup96-SNAP cells were purchased from Cytion (clone 195, #300174 and #300444). The cells were grown at 37 °C in a humidified incubator containing 5% CO2 in McCoys 5A medium (Fisher Scientific) supplemented with 10% fetal bovine serum (FBS) and 1% Antibiotic-Antimycotic solution (Corning, #30–004-CI). For seeding on coverslips, cells were washed with PBS and incubated with Trypsin-EDTA (0.25%) for 3 min until detached. Trypsin was neutralized with fresh medium, and a total of 4 × 10^4^ cells were plated on 1.5H glass coverslips (Menzel-Gläser 22 × 22 mm^2^) and allowed to grow for 2 to 3 days before fixation. For fixation, culture medium was removed, and cells were incubated for 10 min at room temperature (RT) in PEM (80 mM PIPES, 5 mM EGTA, 2 mM MgCl_2_) supplemented with 4% formaldehyde (FA) (32% ampules, Electron Microscopy Sciences, #15714) and 2% sucrose.^33^ Cells were washed 3 times with PBS (Sigma, P4417).

#### Cultured Cell Sample Preparation

Coverslips were attached to custom open-top Perspex chambers using a silicon fast-curing resin. Fixed U-2 OS-Nup96-mEGFP cultured cells were then permeabilized with 0.1% Triton X-100 (Sigma, #T9284) in PBS for 10 min at room temperature. After an exchange of buffer to PBS, cells were incubated in Massive Photonics antibody incubation solution for up to 1 h. The samples were subsequently incubated with 1:200 anti-GFP single-domain nanobody (harboring either Docking Site 3 or custom D2 sequences) for 1 h at room temperature in Massive Photonics incubation solution. Cells were washed 3 times (∼5 min each) with DNA-PAINT buffer (PBS supplemented with an additional 500 mM NaCl, Sigma, S7653). Gold nanoparticles (BBI Solutions, EM.GC150/4) were introduced to the sample 1:1 in DNA-PAINT buffer and allowed to settle onto the coverslip. After sufficient levels of attachment (determined by monitoring the sample on the MINFLUX system and dependent on the sample, aiming for >5 gold particles on the glass surface local to desired imaging regions), samples were washed in DNA-PAINT buffer to remove unbound and jittery gold particles. Occasionally, an additional washing step using 10 mM MgCl_2_ in PBS was used to help remove inadequately bound gold particles that remained on top of cells. Imager 3 was introduced to the sample in a concentration of ∼1 nM diluted in DNA-PAINT buffer.

For the repeat-domain experiments, the labeling was conducted as above, but with the addition of incubating the sample with approximately 200 nM 10× RD P1 for 10 min at room temperature. The samples were washed several times with DNA-PAINT buffer in order to reduce surplus docking sites. Imaging was then conducted with an order of magnitude reduction in imager concentration (∼0.1 nM) in comparison to experiments without the repeat-domains.

#### Photoswitching Labeling

For prefixation, culture medium was removed, and cells were incubated for 30 s at RT with fixation solution consisting of 2.4% Formaldehyde solution (Thermo, 28908) in PBS (Gibco, 14040). Cells were washed once with PBS and permeabilized with 0.4% Triton X-100 (Sigma, T9284) in PBS for 3 min at RT. After another wash with PBS, cells were fully fixed for 30 min at RT with fixation solution, followed by another wash. Samples were then quenched for 5 min at RT with 50 mM NH4Cl (Sigma, 213,330) in PBS, followed by three additional 5 min washes with PBS. To avoid unspecific binding of the labeling agent to the sample, a 30 min incubation at RT with ImageIT Signal Enhancer (Invitrogen, I36933) was performed. Labeling was achieved by incubating the sample for 1 h at RT with a 1 μM solution of SNAP-Surface Alexa-647 (NEB, S9136S) diluted in PBS supplemented with 0.5% BSA (Sigma, A9647) and 1 mM DTT (Sigma, D9779). A final round of three washes of 5 min each at RT with PBS was performed prior to sample mounting.

Gold nanoparticles (BBI Solutions, EM.GC150/4) were introduced to the sample undiluted and allowed to settle onto the coverslip for 5 min at RT. After attachment, samples were washed with PBS several times to remove any unbound gold particles.

Freshly prepared GLOX buffer consisting of 50 mM Tris/HCl (Invitrogen, 15568–025), 10 mM NaCl (Sigma, 71380), 10% (w/v) Glucose (Sigma, G5400), 64 μg/mL catalase (Sigma, C1345) and 0.4 mg/mL glucose oxidase (Sigma, G2133) was adjusted to pH 8.0 and supplemented with 15 mM cysteamine (Sigma, 30070). Samples were then mounted on chambered slides (BMS, 12290) filled with approximately 100 μL of imaging buffer and secured with silicone glue (Picodent eco-sil).

### Data Acquisition

#### Microscope Setup

MINFLUX data were acquired using a commercial 3D MINFLUX microscope (Abberior Instruments) equipped with 405, 485, 561, and 642 nm laser lines. Additionally, a 980 nm IR laser and a PI nano XYZ Stage were used for active sample stabilization. Acquisitions were performed using a 100× 1.45 NA UPlanXAPO oil objective (Olympus), and the pinhole was set to 0.83 AU. The 642 nm laser had a measured power of ∼5.5 mW directly before the periscope. MINFLUX data was acquired using a nominal power setting of 10% which equated to ∼65 μW at the sample plane during the first iteration, this increases 6-fold for the final iteration as per the sequence file, see Supporting Table T7. Power measurements at the sample plane were determined using a slide-sensor from Thorlabs (S170C) placed in the sample holder of the stage.

Fluorophores were excited using the 642 nm laser, and fluorescence was detected via two APDs with different spectral windows: Cy5 near (650–685 nm), and Cy5 far (685–720 nm). The default 3D Imaging sequence provided by Abberior was used to determine the localizations of the fluorescent molecules. This sequence defines the parameters of each iterative localization process, such as the pattern type, the diameter of the targeted coordinate pattern (TCP), and the minimal number of photons required for valid localization, see also (Supporting Table T7).

#### MINFLUX PSF Check and Alignment

Before each acquisition, PSF and pinhole alignment were checked. The red beads from a nanoparticle gold (150 nm) + 2C fluor (120 nm) slide (Abberior #NP-3012) were used to assess the PSF shape. Briefly, 2D and 3D donut-shaped patterns were examined to verify the presence of a zero-intensity center in the *x*–*y*, *x*–*z*, and *y*–*z* planes. Additionally, the donut homogeneity was assessed visually. Pinhole alignment was assessed using red beads, ensuring that the 3D PSF would not be clipped when setting the pinhole to the experimental settings (0.83 AU).

#### MINFLUX Localization Procedure

The localization of a fluorophore during a 3D MINFLUX acquisition is based on an iterative process. The excitation laser, shaped by a spatial light modulator, is scanned along nodes of a search grid, a process termed “scouting”. Once a potential “target” fluorescent marker is detected, the beam is scanned using a specific set of coordinates referred to as the “Target Coordinate Pattern” (TCP) to estimate the position of the fluorophore. The scanning of a TCP is repeated over 9 further iterations (in 3D MINFLUX), decreasing the TCP diameter and increasing the illumination power, to refine this estimate as it progresses to the final iteration.

A final localization of the fluorophore is determined once the ninth iteration has been completed, the procedure is then repeated from iteration 6 to obtain a series of localization estimates within a single “trace”. This process continues until the single-molecule event stops emitting photons or some other condition is met as described below. A trace is retained in postprocessing so long as it includes at least 4 localizations.

Importantly, the transition between iteration *n* to *n* + 1 requires the fulfilment of several criteria. These criteria are based on comparisons between data obtained during the TCP probing and predefined threshold values encoded in a. JSON file referred to as a “sequence” file, see Supporting Table T7 for the full list of values used. These threshold values include: (1) a comparison of the fluorescent molecule emission rate (effective frequency at offset, efo) to the background emission frequency (fbg); (2) a minimal number of photons (phtLimit) collected (effective counts at offset, eco) during the TCP probing (dwell time); (3) a maximal period for which the emission frequency remains below the background (maxOffTime); (4) possibly a center frequency ratio (CFR, parameter ccrLimit) check which represents the ratio between the effective frequency at the TCP center (efc) and the efo. The CFR check is aimed at detecting more than one dye molecule emitting within the PSF region. If the CFR limit is tested for an iteration, then it can exceed the limit a number of times, given by the parameter “stickiness”, before an abortion flag is set. The organization of these criteria are shown in Supporting Figure S2. Once an abortion criterion is met, typically meaning that a single molecule has ceased emitting sufficient photons for detection or a CFR test failed, the current estimation is aborted and leads to the search phase (“scouting”) beginning again from the next node on the search grid.

The MINFLUX imaging sequence used in this study required a minimum of 1060 photons to be collected for the first valid localization (iterations 0–9) and traces were only analyzed if they contained 4 or more localizations adding ∼250 photons per relocalization (iterations 6–9) giving a theoretical minimum of 1810 photons per trace. Further filtering was applied to the localizations in PYME, keeping the data with a CFR value <0.75 and localization errors <15 nm.

#### DNA-PAINT MINFLUX Experiments

Confocal scans were collected prior to MINFLUX acquisitions to capture the diffraction-limited distribution of NPCs using a 488 excitation laser. A qualitative assessment of imager binding kinetics was performed by recording an *xyt* sequence of 50–100 frames for a 3 × 3 μm^2^ region of interest (ROI) with 30 μs dwell time and 30 nm pixel size. These settings enabled clearly visualized circular spots on the scans as imagers were immobilized at the docking sites, see Supporting Media M1. Using the visual display, ROIs were selected for ∼1.5 × 1.5 μm^2^ (small), ∼3 × 3 μm^2^ (medium), or ∼5 × 5 μm^2^ (large) boxed areas for MINFLUX scanning. Depending on the ROI size (small, medium, large) the experiment was conducted for 3, 9, or 12 h respectively. For all experiments the MINFLUX beamline monitoring (MBM) tracking module was used on selected gold particles. Each experiment aimed to have at least 5 gold particles tracked for the duration of the acquisition. The Abberior Instruments 642 nm laser was nominally operated at a software setting of ∼10% achieving ∼65 μW for the first iteration.

#### Photoswitching MINFLUX Experiments

Confocal scans were collected prior to MINFLUX acquisitions to capture the diffraction-limited distribution of NPCs using a 640 excitation laser and the Cy5 near and Cy5 far detectors (total detection window: 650–720 nm). Using the visual display, ∼3 × 3 μm^2^ (medium) ROIs were selected for MINFLUX scanning. Prior to the measurement, off-switching of each ROI was performed by scanning with high laser power (software setting of ∼10–20%, measured power of ∼15–20 μW at the sample) and slow scanning speed (pixel size of 10 nm and dwell time of 5 μs) until only sparse diffraction limited events were observed.

For all experiments the MINFLUX beamline monitoring (MBM) tracking module was used on selected gold particles. Each experiment aimed to have at least 5 gold particles tracked for the duration of the acquisition. The Abberior Instruments 640 nm laser was nominally operated at a software setting of ∼6% power with a recorded measurement of ∼7 μW at the sample. 405 nm activation laser power was gradually increased during the acquisition to ensure a consistent rate of detections while maintaining the single-molecule detection regime.

### Data Analysis and Visualization

Exported. npy or. zarr format data from the Abberior software “Imspector” were imported into the open-source Python Microscopy Environment (PyME) visualization and analysis software, http://github.com/python-microscopy/python-microscopy, with additional functional plug-ins from https://github.com/csoeller/PYME-extra. Upon import into PYME a foreshortening factor of 0.72 was applied to all z coordinates, similar as done previously.^34^ This corrected the separation between nucleoplasmic and cytoplasmic rings from 69.3 ± 7.2 to 50.8 ± 5.8 nm, Supporting Figure S12. MBM tracks were plotted to inspect their agreement with one another and divergent tracks were rejected. To reduce poor localizations and also to reject likely multiemitter events from overlapping emissions, CFR and EFO filtering were conducted, as described previously. Nonspecific labeling of the sample, as previously reported,^13,17^ can still be detected as a true event and was therefore considered for threshold choice in determining NPC labeling, see below. Localizations from the same trace were coalesced to a single detection based on the inherent “traceID” property, as described previously.^9,10^ The full data set was rendered as a 20 nm pixel size Gaussian rendered image^35^ to identify and create a mask of super-resolved NPCs. This mask was then injected back into the PyME pipeline to obtain events within the mask and each NPC was assigned a unique “objectID” to mark these events.

### Quantitative Determination of ELE by Maximum-Likelihood Fitting of NPC Templates

#### NPC Mask Creation

Using the full duration experiment, drift-corrected localization events were rendered as 2D Gaussian images with 10 nm pixel size and saved as. tif files. These images were imported into the software package Fiji, ImageJ,^36^ using Bioformats.^37^ The circular selection tool was used to semiautomatically select identifiable NPCs (excluding NPCs that are partially clipped at MINFLUX ROI edges), recorded using the “ROI Manager” tool and saved as. zip file. In PyME, using the previously rendered image, the imported Fiji ROI were used to create a mask of the NPC locations and subsequently created unique object identifiers (IDs) to tag all events associated with NPCs in the mask.

#### NPC Analysis

For each NPC the associated set of localizations (identified by the unique ID label) were fit to a 3D double ring template using a maximum-likelihood algorithm following an approach described previously.^22^ The 3D template was generated from a geometric arrangement of two rings (nominal diameter 107 nm) spaced 50 nm apart axially. These were convolved with a Gaussian (sigma = 5 nm) in 3D to provide a smooth alignment template. A small, uniformly distributed probability was added to the whole template volume data set to account for background localizations.^22^ We adopted a “smooth” model versus a “detailed” NPC template for robust parameter estimation.^22^ Similarly to previous work, also for robustness, we do not attempt to resolve the pairs of nearby Nup96 sites (laterally ∼ 12 nm apart) in the 8-fold symmetry NPC structure, but rather test for labeling of each of the 8 radial “segments” that each contain these site pairs.^18^ Since we have 3D data, we do this separately for nucleoplasmic and cytoplasmic sides of the NPC, i.e., checking for labeling of 8 nucleoplasmic and 8 cytoplasmic segments, allowing for labeling of up to 16 segments in total per NPC.

For each NPC and its associated localizations the negative logarithm of the likelihood (NLL) is minimized, as described,^22^ to maximize the likelihood. To minimize the NLL, 7 parameters are varied which describe coordinate transformations to line up the MINFLUX localizations with the template, using a global minimizer (the ‘basinhopping’ function of the “scipy.optimize” package), as described^10^ and as illustrated in Figure 2b.

#### Segment Analysis

To check for segment labeling, a threshold of 1 or 3 compound localizations was used to regard a segment as labeled (see threshold determination below). Prior to segment labeling analysis, the aligned NPC data was additionally rotationally aligned to “segment boundaries” as described.^10^

#### Effective Labeling Efficiency

To determine the ELE value for a MINFLUX 3D data set, the template fitting procedure and segment labeling testing were conducted for each NPC in the data set. The data were then pooled to generate a histogram of the number of segments that were labeled across the data set, as shown in Figure 2e, displaying the relative frequency of 0 to 16 segments being detected as labeled. Finally, for obtaining the ELE value, the probabilistic model described in the supplementary model was fit to the data histogram in cumulative form, as shown in Figure 2f. The best fit parameter *p*_LE_ of the model, together with its uncertainty Δ*p*_LE_, are then provided as an estimate of ELE for the given data set.

The complete NPC fitting and ELE estimation procedure is implemented in the “PYMEcs.Analysis.NPC” module of the “PYME-extra” package. A graphical interface is provided by the “NPCcalcLM” module, also part of the “PYME-extra” package, as a plugin for PYMEVisualize.

#### Gallery Mode, Averaging NPCs

To produce an “average” image or a gallery of NPCs from a data set, all normalized coordinates obtained from fitting to the NPC template and rotational segment alignment were overlaid in PYMEVisualize (using the “Add NPC Gallery” command) where (a) it could be chosen to overlay all events in a single coordinate system at the origin or (b) NPCs and/or nucleoplasmic/cytoplasmic segments were arranged next to each other on a regular grid. Functionality (a) was used to generate an average image of all aligned NPC data by rendering all overlaid NPC localizations using Gaussian rendering (pixel size 2 nm, Gaussian standard deviation 2 nm, e.g., images in Figure 4ai, (ii). Functionality (b) was used to generate a gallery of NPCs next to each other for visual inspection with segment boundaries overlaid (e.g., the NPC sample shown in Figure 2e, displaying nucleoplasmic and cytoplasmic localizations side by side for visual inspection).

#### Fourier Shell Correlation Measurements

The 3D MINFLUX data was split into two blocks using the “time blocking” functionality of PYMEVisualize and rendered as separate Gaussian-generated volume images using a Gaussian width of 2 nm with a voxel size of 2 or 3 nm. These were then saved as MRC format files using functionality in PYME-extra (“Save MRC volumes for FSC”) and uploaded to the online Electron Microscopy Data Bank FSC server (https://www.ebi.ac.uk/emdb/validation/fsc/, accessed 14/05/25). The downloaded. xml result files were then processed for plotting using a Jupyter Python notebook.

#### Background and Threshold Determination

Background localizations above and below the NPC focal plane were used to determine the background contribution to our ELE analysis and assist in setting the threshold value. Background localizations from single-domain experiments were analyzed with the same NPC mask used for the specific data set, but with the NPC localizations removed and only using the background localizations. The change in ELE (ΔELE) was determined by purposely shifting the background events into the NPC localization domain by shifting their *z*-coordinates into the NPC region and subsequently subtracting the obtained ELE value with these additional background localizations from the previously obtained ELE values without these additional background events. For standard DNA-PAINT markers, these values were evaluated for a threshold of 1 compound event and indicated very small contributions from background localizations Supporting Figure S3a,b. The repeat DNA-PAINT experiments used an ∼order of magnitude reduced imager concentration leading to a reduced background making estimates for background-only measurements difficult. The background localizations from relatively homogeneous data sets were taken from above and below the NPCs, and their coordinates were axially transformed into the NPC signal. The ΔELE was then measured as described above using a threshold of 1 compound localization and 3 compound localizations per segment, Supporting Figure S3c,d.

#### Further Analysis

Custom Jupyter (https://jupyter.org) notebooks written in python 3.7.13 code were used to create the plots throughout this manuscript and Supporting Information, see data availability comment.

### Statistics and Reproducibility

Data is given as the mean values ± standard deviation. All *p*-values were obtained using the Python library package SciPy (version 1.3.0) and the “stats” module by running independent *t* tests using the function ‘ttest_ind’. A p-value <0.05 was considered as statistically significant and represented on figures as *, *p* < 0.01 as **, and *p* < 0.001 as ***, *ns* denoted nonsignificance.

## Supplementary Material





## Data Availability

The data, the analysis notebooks used to create the figures, example PyME analysis and the MINFLUX imaging sequence used in this study can be accessed from the following Figshare link: 10.6084/m9.figshare.29109266. Opensource PyME installation can be obtained from this repository: https://github.com/csoeller/PYME-test-env. All other data is available upon reasonable request.

## Notes

Supporting Methods for a simple model of ELE in DNA-PAINT; supporting methods for a probabilistic model for Nup96 labeling of NPC structures; MINFLUX concepts; simplified decision tree for a MINFLUX iteration; background measurements and threshold determination for ELE measurements; mean combined site visits for SD DNA-PAINT for a large ROI; photoswitching Alexa Fluor 647 MINFLUX data; MINFLUX event localizations over time; FSC measurements for 3D MINFLUX for SD and RD DNA-PAINT; ELE measurements for repeat DNA-PAINT using a basic threshold of 1; NPC site-loss over time; visits to nucleoplasmic sites versus cytoplasmic sites; Monte Carlo model of DNA-PAINT revisits at target sites versus cytoplasmic sites; Monte Carlo model of DNA-PAINT revisits at target sites; and foreshortening correction measurements; Table of results for: SD DNA-PAINT ELE measurements for different MINFLUX scan sizes acquired over time; SD DNA-PAINT interval measurements for different MINFLUX scan sizes; Alexa Fluor 647 ELE measurements; RD DNA-PAINT ELE measurements for different MINFLUX scan sizes acquired over time using an NPC analysis threshold of 3; RD DNA-PAINT ELE measurements for MINFLUX scan sizes acquired over time using an NPC analysis threshold of 1; RD DNA-PAINT ELE interval measurements for different MINFLUX scan sizes using an NPC analysis threshold of 3; Table containing the 3D MINFLUX imaging sequence parameters (PDF)
Confocal scans demonstrating DNA-PAINT binding events (AVI)

## References

[1] Balzarotti F., Eilers Y., Gwosch K. C. (2017). Nanometer resolution
imaging and tracking of fluorescent molecules with minimal photon
fluxes. Science.

[2] Hell S. W., Wichmann J. (1994). Breaking the diffraction
resolution limit by stimulated
emission: stimulated-emission-depletion fluorescence microscopy. Opt. Lett..

[3] Klar T. A., Jakobs S., Dyba M., Egner A., Hell S. W. (2000). Fluorescence
microscopy with diffraction resolution barrier broken by stimulated
emission. Proc. Natl. Acad. Sci. U.S.A..

[4] Rust M. J., Bates M., Zhuang X. (2006). Sub-diffraction-limit
imaging by
stochastic optical reconstruction microscopy (STORM). Nat. Methods.

[5] Betzig E., Patterson G. H., Sougrat R. (2006). Imaging Intracellular
Fluorescent Proteins at Nanometer Resolution. Science.

[6] Masullo L. A., Lopez L. F., Stefani F. D. (2022). A common framework for single-molecule
localization using sequential structured illumination. Biophys. Rep..

[7] Dempsey G. T., Vaughan J. C., Chen K. H., Bates M., Zhuang X. (2011). Evaluation
of fluorophores for optimal performance in localization-based super-resolution
imaging. Nat. Methods.

[8] Jungmann R., Steinhauer C., Scheible M. (2010). Single-Molecule Kinetics
and Super-Resolution Microscopy by Fluorescence Imaging of Transient
Binding on DNA Origami. Nano Lett..

[9] Ostersehlt L. M., Jans D. C., Wittek A. (2022). DNA-PAINT MINFLUX nanoscopy. Nat. Methods.

[10] Clowsley, A. H. Analysis of RyR2 distribution in HEK293 cells and mouse cardiac myocytes using 3D MINFLUX microscopy bioRxiv 2023 10.1101/2023.07.26.550636.

[11] Jungmann R., Avendaño M. S., Woehrstein J. B. (2014). Multiplexed 3D cellular
super-resolution imaging with DNA-PAINT and Exchange-PAINT. Nat. Methods.

[12] Blumhardt P., Stein J., Mücksch J. (2018). Photo-Induced Depletion
of Binding Sites in DNA-PAINT Microscopy. Molecules.

[13] Clowsley A. H., Kaufhold W. T., Lutz T. (2021). Repeat DNA-PAINT suppresses
background and non-specific signals in optical nanoscopy. Nat. Commun..

[14] Clowsley A. H., Kaufhold W. T., Lutz T. (2020). Detecting Nanoscale
Distribution of Protein Pairs by Proximity-Dependent Super-resolution
Microscopy. J. Am. Chem. Soc..

[15] Brockman J. M., Su H., Blanchard A. T. (2020). Live-cell super-resolved PAINT imaging
of piconewton cellular traction forces. Nat.
Methods.

[16] Chung K. K. H., Zhang Z., Kidd P. (2022). Fluorogenic DNA-PAINT
for faster, low-background super-resolution imaging. Nat. Methods.

[17] Lučinskaitė E., Bokhobza A. F. E., Stannard A. (2024). Reduced Non-Specific
Binding of Super-Resolution DNA-PAINT Markers by Shielded DNA-PAINT
Labeling Protocols. Small.

[18] Thevathasan J. V., Kahnwald M., Cieśliński K. (2019). Nuclear
pores as versatile reference standards for quantitative superresolution
microscopy. Nat. Methods.

[19] Marin, Z. ; Ries, J. Evaluating MINFLUX experimental performance in silico bioRxiv 2025 10.1101/2025.04.08.647786. PMC12783130 41353189

[20] Schindelin J., Arganda-Carreras I., Frise E. (2012). Fiji: an open-source
platform for biological-image analysis. Nat.
Methods.

[21] Marin Z., Graff M., Barentine A. E. S. (2021). PYMEVisualize: an open-source
tool for exploring 3D super-resolution data. Nat. Methods.

[22] Wu Y.-L., Hoess P., Tschanz A. (2022). Maximum-likelihood model
fitting for quantitative analysis of SMLM data. Nat. Methods.

[23] Wade O. K., Woehrstein J. B., Nickels P. C. (2019). 124-Color Super-resolution
Imaging by Engineering DNA-PAINT Blinking Kinetics. Nano Lett..

[24] Schueder F., Stein J., Stehr F. (2019). An order
of magnitude
faster DNA-PAINT imaging by optimized sequence design and buffer conditions. Nat. Methods.

[25] Harauz G., van Heel M. (1986). Exact filters for general geometry
three dimensional
reconstruction. Optik.

[26] Lutz T., Clowsley A. H., Lin R. (2018). Versatile
multiplexed
super-resolution imaging of nanostructures by Quencher-Exchange-PAINT. Nano Res..

[27] Strauss S., Jungmann R. (2020). Up to 100-fold speed-up and multiplexing
in optimized
DNA-PAINT. Nat. Methods.

[28] Zheng Q., Jockusch S., Rodríguez-Calero G. G. (2016). Intra-molecular
Triplet Energy Transfer is a General Approach to Improve Organic Fluorophore
Photostability. Photochem. Photobiol. Sci..

[29] Zheng Q., Juette M. F., Jockusch S. (2014). Ultra-Stable Organic
Fluorophores for Single-Molecule Research. Chem.
Soc. Rev..

[30] Zheng Q., Jockusch S., Zhou Z., Blanchard S. C. (2014). The Contribution
of Reactive Oxygen Species to the Photobleaching of Organic Fluorophores. Photochem. Photobiol..

[31] Scheckenbach, M. ; Close, C. ; Bauer, J. Minimally Invasive DNA-Mediated Photostabilization for Extended Single-Molecule and Super-resolution Imaging bioRxiv 2025 10.1101/2025.01.08.631860.

[32] Steen P. R., Unterauer E. M., Masullo L. A. (2024). The DNA-PAINT palette:
a comprehensive performance analysis of fluorescent dyes. Nat. Methods.

[33] Jimenez A., Friedl K., Leterrier C. (2020). About samples,
giving examples: Optimized
Single Molecule Localization Microscopy. Methods.

[34] Gwosch K. C., Pape J. K., Balzarotti F. (2020). MINFLUX nanoscopy delivers
3D multicolor nanometer resolution in cells. Nat. Methods.

[35] Baddeley D., Cannell M. B., Soeller C. (2010). Visualization of localization microscopy
data. Microsc. Microanal..

[36] Schindelin J., Arganda-Carreras I., Frise E. (2012). Fiji: an open-source
platform for biological-image analysis. Nat.
Methods.

[37] Linkert M., Rueden C. T., Allan C. (2010). Metadata matters: access
to image data in the real world. J. Cell Biol..

